# Exploring the Effects of Local Air Pollution on Popliteal Artery Aneurysms

**DOI:** 10.3390/jcm13113250

**Published:** 2024-05-31

**Authors:** Maria Elisabeth Leinweber, Katrin Meisenbacher, Thomas Schmandra, Thomas Karl, Giovanni Torsello, Mikolaj Walensi, Phillip Geisbuesch, Thomas Schmitz-Rixen, Georg Jung, Amun Georg Hofmann

**Affiliations:** 1 FIFOS—Forum for Integrative Research and Systems Biology, 1170 Vienna, Austria; 2 Department of Vascular and Endovascular Surgery, University Hospital Heidelberg, 69120 Heidelberg, Germany; 3 Department of Vascular Surgery, Sana Klinikum Offenbach, 63069 Offenbach, Germany; 4 Department of Vascular and Endovascular Surgery, Klinikum am Plattenwald, SLK-Kliniken Heilbronn GmbH, 74177 Bad Friedrichshall, Germany; 5 Department for Vascular Surgery, Franziskus Hospital Münster, 48145 Münster, Germany; 6 Department of Vascular Surgery and Phlebology, Contilia Heart and Vascular Center, 45138 Essen, Germany; 7 Department of Vascular and Endovascular Surgery, Klinikum Stuttgart, 70199 Stuttgart, Germany; 8 German Society of Surgery, Langenbeck-Virchow-Haus, Luisenstraße 58/59, 10117 Berlin, Germany; 9 Department of Vascular and Endovascular Surgery, Luzerner Kantonsspital, 6000 Lucern, Switzerland

**Keywords:** popliteal artery aneurysm, air pollution, particulate matter, peripheral aneurysm

## Abstract

**Objectives:** A growing body of evidence highlights the effects of air pollution on chronic and acute cardiovascular diseases, such as associations between PM_10_ and several cardiovascular events. However, evidence of the impact of fine air pollutants on the development and progression of peripheral arterial aneurysms is not available. **Methods:** Data were obtained from the multicenter PAA outcome registry POPART and the German Environment Agency. Means of the mean daily concentration of PM_10_, PM_2.5_, NO_2,_ and O_3_ concentrations were calculated for 2, 10, and 3650 days prior to surgery for each patient. Additionally, weighted ten-year averages were analyzed. Correlation was assessed by calculating Pearson correlation coefficients, and regression analyses were conducted as multiple linear or multiple logistic regression, depending on the dependent variable. **Results:** For 1193 patients from the POPART registry, paired air pollution data were available. Most patients were male (95.6%) and received open surgical repair (89.9%). On a regional level, the arithmetic means of the daily means of PM_10_ between 2000 and 2022 were neither associated with average diameters nor runoff vessels. Negative correlations for mean PAA diameter and mean NO_2_, as well as a positive correlation with mean O_3_, were found; however, they were not statistically significant. On patient level, no evidence for an association of mean PM_10_ exposure over ten years prior to inclusion in the registry and PAA diameter or the number of runoff vessels was found. Weighted PM_10,_ NO_2,_ and O_3_ exposure over ten years also did not result in significant associations with aneurysm diameter or runoff vessels. Short-term air pollutant concentrations were not associated with symptomatic PAAs or with perioperative complications. **Conclusions:** We found no indication that long-term air pollutant concentrations are associated with PAA size or severity, neither on a regional nor individual level. Additionally, short-term air pollution showed no association with clinical presentation or treatment outcomes.

## 1. Introduction

Besides many known modifiable (smoking, hypertension) and non-modifiable (age, sex, ethnicity) risk factors, increasing evidence highlights the impact of ambient air pollution on chronic and acute cardiovascular conditions [1,2,3,4]. The most commonly studied air pollutants with respect to their effects on human health include ozone, carbon monoxide, sulfur dioxide, nitrogen oxides, but also particulate matter, which can be divided into particles with an aerodynamic diameter of <10 μm (PM_10_), finer particles with an aerodynamic diameter of <2.5 μm (PM_2.5_), particles between 2.5 and 10 μm (PM_coarse_), and ultrafine particles (UFP), which measure less than 0.1 μm [1]. Sources of particulate matter can include emissions from domestic energy use (e.g., heating and cooking), which are most prevalent in countries such as India and China, as well as emissions from transport, power generation, and agriculture, which make the largest relative contribution to PM_2.5_ in regions such as Europe, East Asia, and the eastern United States [5,6]. In Europe alone, it is estimated that the annual excess mortality due to air pollution could be more than 790,000, of which 40–80% could be due to cardiovascular events [6]. Associations between short- and long-term exposure to PM_2.5_ and PM_10_ and several cardiovascular events have been identified, including pulmonary embolism [7,8], stroke [9,10], myocardial infarctions [11,12], congestive heart failure, arrhythmias [2,13,14], and acute aortic dissections [15,16,17]. Air pollution is also associated with cardiovascular risk factors such as arterial hypertension and the progression of arteriosclerotic lesions [18,19]. Additionally, both in murine models and in a large population-based study, associations with aortic aneurysm genesis and progression have been described [20,21]. For example, the recent analysis of 449,463 participants from the UK Biobank showed that long-term exposure to PM_2.5_, PM_10_, nitrogen dioxide, and nitrogen oxides was associated with an increased risk of incident abdominal aortic aneurysms, with a persistent effect even in participants with low exposure [21].

While the underlying pathophysiology of air pollution’s effects on the cardiovascular system is not fully understood, oxidative stress, inflammation, and endothelial dysfunction induced by fine air pollutants have been suggested as mechanisms [22,23,24,25,26].

Although the Lancet Commission on Pollution and Health recognized the importance of air pollution to general health and called for further research to fill knowledge gaps, there are limited data on the effect of ambient air pollution and aneurysmatic diseases [27]. Whereas associations of air pollutants and aortic aneurysms and dissections have been described previously [9,15,20,21,28], to the best of the authors’ knowledge, no evidence on the impact of air pollution on the development and progression of peripheral arterial aneurysm exists. Popliteal artery aneurysms (PAA) are the most common peripheral aneurysms but are overall rare, with a prevalence of only 1% in elderly men [29,30]. The POPART Registry represents a multi-center depiction of open and endovascular PAA procedures in Germany, and its main results have been recently published [31].

In this analysis, data on associations between particulate matter pollutants PM_10_ and PM_2.5_, as well as gaseous air pollutants like O_3_ and NO_2_ on diameter, quality of runoff vessels, and clinical presentation of popliteal artery aneurysms will be presented.

## 2. Methods

### 2.1. IRB Approval

POPART is approved by the Ethics Committee of the University Hospital Frankfurt (approval no. 218/4). POPART is also listed in the German Registry of Clinical Studies (identification no. DRKS00017609).

### 2.2. Design

This study design has been described before [31,32]. POPART is a multicenter outcome registry for endovascular and open PAA repair, with more than 42 centers in Germany and Luxembourg. Participating centers are required to offer both open and endovascular PAA repair. All patients aged >18 years who have presented with a PAA to one of the participating centers since 2010 are eligible for enrollment. Before study inclusion, patients had to provide informed and written consent. As a non-interventional study, the indication for treatment as well as the follow-up protocol were solely at the discretion of the attending surgeon and unrelated to study participation. Data entry is conducted via an electronic case report form.

### 2.3. Data

Data regarding PM_10_, PM_2.5_, NO_2_, as well as O_3_ concentrations (all mcg/m^3^), were provided by the Umweltbundesamt (German Environment Agency, Dessau-Roßlau, Saxony-Anhalt, Germany). The dataset included mean daily concentrations from January 2000 to December 2022. Monitoring stations were paired with the city of the respective center. In cases where the location of the center had no monitoring station, a proxy station was selected. (Supplementary Table S1) We calculated arithmetic means of the mean daily concentration for 2, 10, and 3650 (also referred to as ten-year average) days prior to the surgery date for each patient. While there is no information on the patient’s home address prior to inclusion in the registry, we deemed the analysis strategy nevertheless relevant. Since the demographic features of the study cohort make frequent moving unlikely, long-term air pollution averages should adequately capture exposure, and due to the fact that acute presentations require immediate treatment, short-term exposure should analogously be sufficiently accurate. Additionally, we calculated weighted ten-year averages with a linear distribution of increasing weights until the day of surgery. Missing values were removed. Data regarding PM_10_ and NO_2_ levels were available for 1193 patients, while data regarding O_3_ concentrations were available for 946 patients. Since PM_2.5_ is only rather recently part of the portfolio of most monitoring stations, analyses were only possible in a subset of 411 patients focusing on short-term effects (2 and 10 days).

### 2.4. Analysis

Patient characteristics were analyzed by descriptive statistical methods, including calculation of measures of central tendency and dispersion. Correlation was assessed by calculating Pearson correlation coefficients, including the 95% CI. Regression analyses were conducted as multiple linear or multiple logistic regression, depending on the dependent variable. Covariates included sex, number of runoff vessels, smoking, and age. Statistical significance was determined based on a *p*-value < 0.05 corresponding to a 95% CI, not including 0 or 1 depending on the underlying metric. Concentration differences between groups were assessed using a two-sample t-test. All statistical analyses were performed with R version 4.1.3 (R Foundation for Statistical Computing, Vienna, Austria) in RStudio (Posit PBC, Boston, MA, USA).

## 3. Results

### 3.1. Sample Characteristics

The total study sample included 1193 patients from the POPART registry, where paired air pollution data were available. The study cohort consisted of 4.4% female and 95.6% male patients. PAAs were equally distributed among the left and right lower extremities with a median diameter of 27 mm. Arterial hypertension was the most prevalent comorbidity (67.4%), and approximately a third of patients were smokers. About a third of all patients had a concomitant abdominal aortic aneurysm. Regarding clinical presentation, 15.5% of patients entered the registry with acute limb ischemia related to their PAA. After endovascular treatment, 5.3% had a recorded complication, while 16.8% of patients receiving open surgical repair had a minor or major perioperative complication. N = 33 patients (2.8%) were treated conservatively. Demographic and clinical characteristics are shown in Table 1.

### 3.2. Ecological Associations

**Figure 1:**
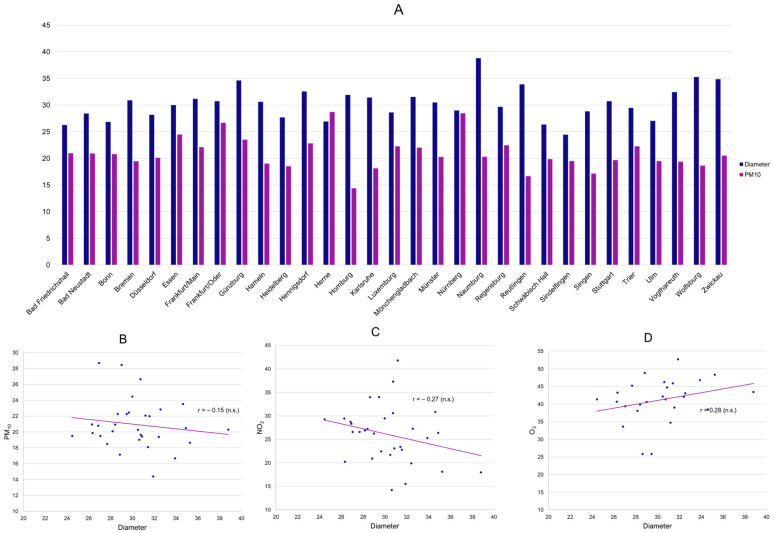
Mean PM_10_ (mcg/m^3^) and mean PAA diameter (mm) per center (**A**) as well as displayed in a scatter plot, including a linear trendline (**B**) (n.s.: 95% CI of correlation coefficient r includes 0). Scatter plots of mean NO_2_ (**C**) as well as mean O_3_ (**D**) and mean PAA diameter per center (n.s.: 95% CI of correlation coefficient r includes 0).

The designed multi-stage analysis plan included both an investigation on a (regional) population level (ecological study) as well as an investigation using individual patient data. To investigate whether higher levels of air pollution were associated with a more advanced disease burden in popliteal artery aneurysms on a regional level, we assessed if higher concentrations of PM_10_ correlated with aneurysm diameters or runoff vessels at each center/monitoring station. The arithmetic means of the daily means of PM_10_ between 2000 and 2022 were neither associated with average diameters (Figure 1A,B) nor runoff vessels. The crude association analysis was further validated by performing a linear regression analysis for both diameter and runoff vessels at each center using PM_10_, the proportion of female patients, age, and the proportion of smokers at each center as covariates. Congruent to the crude analysis, PM_10_ showed no association with diameters or runoff vessels at a regional level. Similar inconclusive findings were obtained for the remaining air pollutants, where negative correlations for mean PAA diameter and mean NO_2_, as well as a positive correlation with mean O_3,_ were found, but not at a statistically significant level (Figure 1C,D).

### 3.3. Patient-Level Analysis

**Figure 2:**
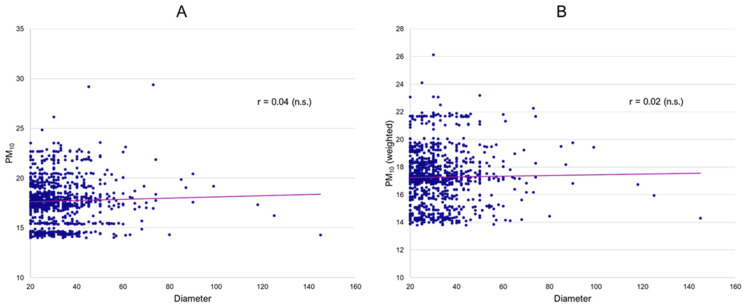
Association of PAA diameter and 10-year mean PM_10_ (**A**) and weighted 10-year mean PM_10_ (**B**) (all mcg/m^3^).

Subsequently, individual patient data were investigated. There was no conclusive evidence for an association of mean PM_10_ exposure over ten years (long-term effects) prior to inclusion in the registry and popliteal artery aneurysm diameter (Figure 2A) or number of runoff vessels. (Table 2) Analogous to the ecological analyses, both a crude correlation analysis as well as regression models, including relevant covariates (sex, number of runoff vessels, smoking, and age), were conducted. However, again, no significant association was obtained. Similarly, weighted PM_10_ exposure over ten years, where more recent daily mean concentrations had a higher impact on the calculated average (long-term effects with increasing importance of more recent exposure), also did not result in significant associations with aneurysm diameter (Figure 2B) or runoff. The fact that weighted ten-year averages are consistently lower reflects a general decrease in particulate matter concentrations in Germany since 2000, corresponding with large-scale environmental analyses. Similar analyses for NO_2_ as well as O_3_ also showed no indication of any effects on PAA diameter or runoff. (Table 2) In a sensitivity analysis, we investigated whether long-term mean air pollutant exposure was associated with concomitant abdominal aortic, iliacal, and contralateral popliteal aneurysms, as well as intraluminal thrombus. However, no significant differences in ten-year concentration means were found between the groups (all *p*-values > 0.05).

### 3.4. Effect of Air Pollution on Symptoms and Outcomes

**Figure 3:**
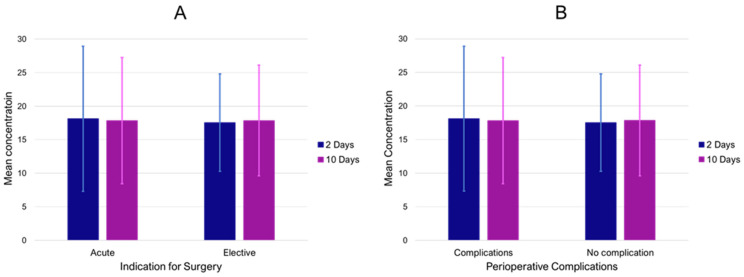
Mean PM_10_ concentrations (mcg/m^3^) 2 and 10 prior to inclusion in the registry for acute and elective cases (**A**) as well as in surgical patients with and without perioperative complications (**B**). Bars indicate standard deviation.

Finally, the effects of short-term exposure (2 and 10 day means) were analyzed regarding clinical presentation and treatment outcomes. There was no significant difference in short-term concentrations of PM_10_ (Figure 3A), PM_2.5_, NO_2_, and O_3_ between PAA patients with and without ALI (all *p*-values > 0.05). Additionally, PM_10_ levels of the index date and lag1–9 were not observed to be associated with acute symptomatic presentations in the registry. There was no further indication that these short-term concentrations prior to surgery were associated with perioperative complications, neither for overall complications (Figure 3B) nor for thromboembolic complications (MI, stroke, and peripheral thrombosis) (all *p*-values > 0.05).

## 4. Discussion

Major air pollution disasters, such as the London fog of 1952 with an estimated 4000 deaths, but also similar episodes in Dublin in 1982 and in the German Ruhr area in 1962, 1979, and 1982, have been important landmarks in epidemiological research to identify air pollution as a relevant factor in excess mortality and general cardiopulmonary morbidity [33]. Despite many improvements in air quality standards, the impact of ambient air pollution on human health and cardiovascular mortality remains an issue of concern [27]. In 2019, an analysis of ambient particulate air pollution and daily mortality in 652 cities from 24 countries showed that an increase of 10 μg per cubic meter in the 2-day moving average of PM_10_ concentration was associated with an increase of 0.36% in daily cardiovascular mortality [34]. Recent analyses have demonstrated that long-term residential exposure to high traffic and air pollutants is associated with several cardiovascular conditions like the degree of coronary artery calcification [35], myocardial infarctions [11,12], acute aortic dissections [15,16,17] and abdominal aortic aneurysm rupture [36]. However, population-based data on the impact of air pollution on the incidence and growth of central or peripheral aneurysms are scarce.

Considering the available evidence on PAAs, their pathophysiology is likely to be multifactorial, including comorbidities, lifestyle, or mechanical properties. Genetic predispositions might be discussed, taking into account the relatively high co-prevalence of other aneurysms, such as abdominal aortic aneurysms. However, both pathologies also share similar risk factors, which limits the quantification of the potential inheritable risk. Nevertheless, these aneurysms feature both clinically and histologically distinct profiles different from aneurysms resulting from inherited congenital dysfunctions of the collagen/elastin tissue of the arterial wall, where adverse arterial events are mostly encountered in relatively young patients, frequently in emergency settings [37]. PAAs are most commonly identified in patients above 60, which indicates that the pathology is a result of long-lasting exposure to risk factors and causal mediators.

In the present work, we aimed to investigate the potential effects of air pollution on PAA, which, to the best of our knowledge, have never been studied before. We did so by following a three-layered approach: first, by focusing on an ecological analysis on a center level; second, by diving into a patient-level analysis; and third, by investigating relationships between clinical presentations as well as outcomes. The ecological association analysis resulted in (very) low correlations between long-term PM_10_, NO_2_ as well as O_3_ concentrations and PAA diameters or runoff. Furthermore, none of these correlations reached statistical significance, and the introduction of potential confounders did not affect the results. The patient-level analyses also resulted in no association between long-term air pollutant concentrations and PAA diameters or runoff. Similarly, short-term concentrations of PM_10_, PM_2.5_, NO_2_, and O_3_ were not associated with emergency presentations or impaired outcomes.

The general pathophysiology of the extrapulmonary effects of air pollutants is not conclusively understood but three main pathways on the cardiovascular system are currently discussed: (a) the release of proinflammatory mediators and vasoactive molecules from lung-derived cells, (b) disturbance of the balance of the systemic autonomic nervous system or cardiac rhythm due to particle interference with different lung receptors or nerves, and (c) possibility of PM or particulate components (organic compounds, metals) entering the circulatory system [23,24,25,26,38,39,40,41].

Although a recent publication has shown that exposure to PM_2.5_ increases the diameter of the thoracic aorta in mouse models [20], the cumulative body of evidence focusing on biological mechanisms related to air pollution and aneurysms is weak. Considering possible synergies between similar pathways in aneurysm development due to systemic vascular dysfunction, inflammation, and atherosclerosis progression, further studies are needed to investigate whether there are biological mechanisms between air pollutants and aneurysm development [42].

Associations between both gaseous and particulate matter pollutants and acute thromboembolic cardiovascular events have been described before [7,8,9,10,11,12]. Several mechanisms by which air pollution induces an activated coagulation state are suggested, including induction of endothelial dysfunction and inflammatory responses, as well as generation of reactive species and alteration of coagulation factors [43,44,45,46,47,48].

However, we did not find significant correlations in specificity or associations in general between short-term air pollutant concentrations and symptomatic presentations. To our knowledge, comparable studies on short-term air pollution and symptomatic events in aneurysms do not exist. Comparing them to other thromboembolic events like stroke, myocardial infarction, or pulmonary embolism is limited due to partially different pathomechanisms and potential differences in methodology. This may be exacerbated by the considerable uncertainty about the risk factors that ultimately contribute to PAA-related thromboembolic events in general [49]. Furthermore, no associations between overall perioperative complications, thromboembolic complications or major cardiovascular events, and short-term air pollution concentrations could be shown. However, it should be noted that the small number of perioperative complications (low event rate) may limit the assessment of possible associations with air pollution.

In order to minimize confounding, we performed multiple regression analyses as an adjunct to the crude (unadjusted) correlation analyses. However, certain potential limitations persist based on the available data in the POPART registry, such as patient address prior to recruitment. As air pollution constitutes a predominantly local environment the exact distance between the monitoring station and the patient’s home address and even work setting as well as environment might impose a general limitation to our results.

This study is further limited by its observational registry design. Treatment indication, preoperative patient, and imaging assessment were at the discretion of the treating surgeon and could not be externally validated. Despite the limited definition of inclusion and exclusion criteria to enable a more comprehensive depiction of the PAA patient population, incomplete screening may have resulted in an analysis of a study population that lacks representativeness.

Furthermore, specific data on aneurysm morphology, medication use prior to hospital admission, treatment adherence, and exposure-related factors, such as patients’ work environment, structural housing situation, and leisure and outdoor activities, were not captured in the electronic case report form. Considering that the average person in the European Union spends 80–90% of the time in indoor environments [50], information on indoor air pollution levels might be very relevant to fully analyze potential effects on health and disease. Although household air pollution is a problem mainly attributed to low-income countries due to the use of solid fuels for heating and indoor cooking, formaldehyde, volatile organic compounds, and semi-volatile organic compounds emitted from building materials and furnishings, household chemicals, as well as indoor tobacco smoke are nevertheless significant air pollutants in European households [50]. Therefore, the mean daily concentrations of air pollutants are only an approximate estimate of actual exposure. These limitations could be addressed in a dedicated case-control study.

Despite the comprehensive knowledge of the effects of short- and long-term exposure to air pollution on human health, the evidence is limited to a selected panel of cardiovascular diseases, as discussed elsewhere [42]. However, it should be noted that such investigations frequently report statistically significant associations that might not correspond to an epidemiologically significant impact (significant but low odds or hazard ratios), as in the case of a recent study on the effects of NO_2_ and peripheral arterial disease [51]. Although air quality standards have improved in recent decades, relevant research projects on this topic have not lost their importance, considering that even at air pollutant concentrations below current European air quality standards, associations with cardiovascular disease have been described [52,53,54].

In our analysis, no indication of any effects on PAA severity, neither on a regional nor on an individual level, regardless of the type of pollutant, was observed. Even when considering the limitations of the present study, it appears reasonable to assume that air pollution does not constitute a major risk factor for PAA on a public health scale in Germany. However, as a dose–response relationship cannot be ruled out on the basis of the currently available evidence, further follow-up studies are needed to assess whether higher exposures to air pollutants (e.g., individual workplace exposures, countries with different air pollution limits) pose a risk for the development of PAA in selected individuals. These studies are needed to assess the generalizability of the results presented in light of the very different air quality standards around the world. For example, while the annual limit value for PM_2.5_ in Australia is 8 μg/m^3^ and 20 μg/m^3^ in the European Union in 2020, China (35 μg/m^3^) and India (40 μg/m^3^) have much higher limit values [55,56]. While the consistency of our results establishes a solid base of evidence, future investigations are both indicated and warranted.

## Figures and Tables

**Table 1:** Patient characteristics. Age and PAA diameter are depicted as median (first–third quartile). ALI—acute limb ischemia, CKD—chronic kidney disease.

	N = 1193
Female:Male	53 (4.4%):1140 (95.6%)
Age	69 (62–77)
Left:Right	595 (49.9%):598 (50.1%)
PAA diameter (mm)	27 (21–35)
Runoff vessels	
none	72 (6.0%)
1	233 (19.5%)
2	377 (31.6%)
3	511 (42.8%)
Hypertension (%)	67.4
Cardiac comorbidity (%)	36.4
CKD (%)	12.3
Diabetes (%)	17.0
Concomitant AAA (%)	32.6
Smoking (%)	32.1
ALI (%)	15.5
Endovascular interventions (%)	11.1
Complications (open surgery) (%)	16.8

**Table 2:** Unadjusted correlation between mean 10-year air pollutant concentration and PAA diameter as well as number of runoff vessels.

	PAA Diameter	Runoff
PM_10_	0.04 (−0.02–0.09)	0.08 (0.02–0.13)
PM_10_ (weighted)	0.01 (−0.04–0.07)	0.08 (0.03–0.14)
NO_2_	0.00 (−0.05–0.06)	0.08 (0.02–0.14)
O_3_	−0.02 (−0.08–0.05)	−0.09 (−0.15–−0.02)

## Data Availability

Data requests have to be directed at the POPART principal investigators.

## Supplementary Materials

The following supporting information can be downloaded at: https://www.mdpi.com/article/10.3390/jcm13113250/s1, Table S1. Stations that were used for data collection. (ID = station ID of the Umweltbundesamt) List of POPART Registry collaborators (in the order of their time of participation).
