# Supplementary material for: Exploring the Effects of Local Air Pollution on Popliteal Artery Aneurysms
Source: J Clin Med. 2024 May 31;13(11):3250. doi: 10.3390/jcm13113250 (PMC11172973; doi:10.3390/jcm13113250)
Supplement: Supplementary file 1 [file jcm-13-03250-s001.zip › jcm-2935626-supplementary.pdf]

Supplementary Table S1. Stations that were used for data collection.(ID = station ID of the *Umweltbundesamt*)

City	Monitoring Station (ID)
**Bad Friedrichshall**	DEBW015
**Bad Neustadt**	DEBY068
**Bonn**	DENW062
**Bremen**	DEHB001
**Bremen-Rotkreuz**	DEHB001
**Düsseldorf**	DENW071
**Essen**	DENW024
**Essen (Krupp KH)**	DENW024
**Frankfurt/ Oder**	DEBB045
**Frankfurt/ Main**	DEHE005
**Günzburg**	DEBY052
**Hameln**	DENI041
**Heidelberg**	DEBW009
**Hennigsdorf**	DEBE010
**Herne**	DENW2023 + DENW243 + DENW203
**Homburg**	DESL018
**Karlsruhe**	DEBW081
**Konstanz**	DEBW052
**Luxembourg**	DERP020
**Ludwigslust**	DEBB063
**Mönchengladbach**	DENW096
**Münster (Franziskus Spital)**	DENW095
**Münster (Universitätsklinik)**	DENW095
**Naumburg**	DETH041
**Nürnberg**	DEBY058
**Regensburg**	DEBY028
**Reutlingen**	DEBW027
**Schwäbisch Hall**	DEBW056
**Sindelfingen**	DEBW042
**Singen**	DEBW052
**Stuttgart (Klinikum)**	DEBW013
**Stuttgart (Karl-Olga K)**	DEBW013
**Stuttgart (Robert-Bosch KH)**	DEBW013
**Trier**	DERP020
**Trier (Mutterhaus)**	DERP020
**Ulm**	DEBW019
**Vogtareuth**	DEBY088
**Wolfsburg**	DENI020
**Zwickau**	DESN091

List of POPART Registry collaborators (in the order of their time of participation):

Kyriakos Oikonomou, MD, PhD, Department of Vascular and Endovascular Surgery, University Hospital Frankfurt am Main, Frankfurt am Main, Germany; Martin Storck, MD, PhD, Department of Vascular and Thoracic Surgery, Klinikum Karlsruhe, Karlsruhe, Germany; Kai Balzer, MD, PhD, Department of Vascular and Endovascular Surgery, St. Marien-Hospital, Bonn, Germany; Ulrich Kugelmann, MD, Department of Vascular and Endovascular Surgery, Kreiskliniken Guenzburg-Krumbach, Guenzburg, Germany; Christina Schneider, MD, Department of General and Vascular Surgery, Krankenhaus der Barmherzigen Brueder Trier, Trier, Germany; Michael Engelhardt, MD, PhD, Department of Vascular and Endovascular Surgery, Bundeswehrkrankenhaus Ulm, Ulm, Germany; Michael Petzold, MD, Krankenhaus Maerkisch Oderland, Strausberg, Germany; Barbara Weis-Mueller, MD, PhD, Kliniken Maria Hilf, Department of Vascular surgery and Angiology, Moenchengladbach, Germany; Markus Wortmann, MD, Department of Vascular, Endovascular and Transplantation Surgery, Klinikum Stuttgart, Stuttgart, Germany; Sebastian Popp, MD, Department of Vascular and Endovascular Surgery, Schoen-Klinik Vogtareuth, Vogtareuth, Germany; Dirk Grotemeyer, MD, PhD, Service de Chirurgie Vasculaire, Hôpital Kirchberg, Hôpitaux Robert Schuman, Luxembourg; Heiner Wenk, MD, PhD, Klinik Lilienthal, Lilienthal, Germany; Roushanak Shayesteh-Kheslat, MD, Department of General, Visceral, Vascular, and Pediatric Surgery, University Hospital Homburg/Saarland, Homburg/Saar, Germany; Giovanni Torsello, MD, PhD, Institut für vaskulaere Forschung, St. Franziskus-Hospital GmbH, Muenster, Germany; Dittmar Böckler, MD, PhD, Department of Vascular and Endovascular Surgery, University Hospital Heidelberg, Heidelberg, Germany; Johannes Hoffmann, MD, PhD, Department of Vascular Surgery and Phlebology, Contilia Herz und Gefaeßzentrum, Essen, Germany; Hubert Schelzig, MD, PhD, Clinic of Vascular and Endovascular Surgery, University Clinic Duesseldorf, Heinrich-Heine-University, Duesseldorf, Germany; Yush Roopa, MD, Department of Vascular and Endovascular Surgery, Klinikum am Plattenwald, SLK-Kliniken Heilbronn GmbH, Bad Friedrichshall, Germany; Thomas Strohschneider, MD, Department of Vascular, Endovascular Surgery and Angiology, Karl-Olga Krankenhaus, Stuttgart, Germany; Thomas Noppeney, MD, PhD, Department of Vascular Surgery, University Hospital Regensburg, Regensburg, Germany; Viktor Reichert, MD, Department of Vascular and Endovascular Surgery, Klinikum Sindelfingen-Boeblingen, Sindelfingen, Germany; Uwe Lorenz, MD, Department of Vascular Surgery, Oberhavel Kliniken, Hennigsdorf, Germany; Karin Pfister, MD, PhD, Department of Vascular Surgery, University Hospital Regensburg, Regensburg, Germany; Shoaeddin Damirchi, MD, Department of Vascular Surgery, Evangelische Krankenhaus Herne, Herne, Germany; Tomislav Stojanovic, MD, PhD, Department of Vascular and Endovascular Surgery, Klinikum Wolfsburg, Wolfsburg, Germany; Alexander Oberhuber, MD, PhD, Department of Vascular and Endovascular Surgery, University Hospital Muenster, Muenster, Germany; Bernd Lobenstein, MD, Department of Vascular Surgery, Klinikum Naumburg, Naumburg, Germany; Tolga Atilla Sagban, MD, Department of Vascular Surgery, Sana Klinikum Hameln-Pyrmont, Hamelin, Germany; Tomas Pfeiffer,

MD, PhD, Department of Vascular and Endovascular Surgery, Hegau-Bodensee-Klinikum Singen, Singen, Germany; Johann Koller, MD, Department of Vascular and Endovascular Surgery, Kreiskliniken Reutlingen, Reutlingen, Germany; Christian Sprenger, MD, Department of Vascular and Endovascular Surgery, Klinikum Mutterhaus der Borromaerinnen, Trier, Germany; Thomas Kruschwitz, MD, Claus-Georg Schmedt, MD, PhD, Department of Vascular Surgery, Diakonie-Klinikum Schwaebisch Hall, Schwaebisch Hall, Germany; Frank Marquardt, MD, Department of Vascular Surgery, Rotes Kreuz Krankenhaus Bremen, Bremen, Germany; Thomas Schmandra, MD, PhD, Department of Vascular and Endovascular Surgery, Rhoen Klinikum, Campus Bad Neustadt, Bad Neustadt, Germany; Dorothee Bail, MD, PhD, Robert-Bosch-Krankenhaus Stuttgart, Stuttgart, Germany.
